# Fentanyl-associated anaphylaxis in an infant with tetralogy of Fallot: a case report

**DOI:** 10.1186/s40981-019-0254-x

**Published:** 2019-05-21

**Authors:** Ayano Teshigawara, Shinichi Nishibe, Saori Horie, Masako Hakone, Yoshihiro Yamaji, Akira Akasawa, Kohichi Yoshida, Emi Morikawa

**Affiliations:** 1 Department of Anesthesiology, Tokyo Metropolitan Children’s Medical Center, 2-8-29 Musashidai, Fuchu, Tokyo 183-8561 Japan; 2 Department of Anesthesiology, Kitasato University Hospital, 1-15-1, Kitasato, Minami-ku, Sagamihara, Kanagawa 252-0375 Japan; 3 Department of Allergology, Tokyo Metropolitan Children’s Medical Center, 2-8-29 Musashidai, Fuchu, Tokyo 183-8561 Japan

**Keywords:** Fentanyl, Intradermal testing, Anaphylaxis, Remifentanil, Morphine

## Abstract

**Background:**

Anaphylactic reactions to opioids are rare. We report a case of an infant who experienced fentanyl-induced anaphylaxis.

**Case presentation:**

A 2-month-old male was scheduled to undergo a Blalock-Taussig shunt. Following uneventful anesthetic induction, he experienced profound hypotension and generalized erythema. Anaphylaxis was clinically diagnosed, and he was treated with epinephrine, vasopressin, and fluids. The surgery was canceled, and he was transferred to the intensive care unit after restoration of his hemodynamic status. Intradermal testing was performed for all of the drugs given during the anaphylactic event on postoperative day (POD) 3. The results showed a positive reaction to fentanyl. For the second anesthesia scheduled on POD 5, morphine sulfate was selected as an alternative opioid. Anesthesia was maintained uneventfully with sevoflurane, morphine, and rocuronium.

**Conclusion:**

Intradermal testing revealed fentanyl anaphylaxis. We were able to manage the patient by using of morphine as an alternative opioid for the subsequent anesthesia.

## Background

Anaphylaxis during anesthesia reportedly occurs at a rate of 0.46/10,000 cases in Japan and is thought to be responsible for cardiac arrest in 0.03/10,000 cases [1]. The most common causes are neuromuscular blocking agents, antibiotics, and latex [2, 3]. In contrast, opioids very rarely trigger perioperative anaphylaxis and account for only 1.3% of all cases. The incidence of fentanyl-induced anaphylaxis is particularly low at less than 0.4% [4].

We report herein a case of an infant with tetralogy of Fallot (TOF) who experienced anaphylaxis after uneventful anesthesia induction for a Blalock-Taussig shunt (BTS) operation. Subsequent intradermal testing identified fentanyl as the causative agent of the anaphylaxis.

## Case presentation

A male infant born at 39 weeks and 5 days of gestation with a birth weight of 2876 g was referred to our hospital due to cyanosis and low peripheral arterial oxygen saturation (SpO_2_; 85–92% on room air). Upon admission, echocardiography showed TOF, and fluorescence in situ hybridization (FISH) confirmed 22q11.2 deletion syndrome. In the next 3 weeks, his SpO_2_ gradually decreased, and anoxic spells became apparent during crying and feeding. Oral propranolol was prescribed to prevent anoxic spells prior to surgery.

When he was 2 months old (57 cm, 4.2 kg), elective BTS creation was scheduled. The patient had no history of general anesthesia. His baseline blood pressure (BP) while in the operating room was 94/49 mmHg, his heart rate (HR) was 144 beats per minute (bpm), and his SpO_2_ was 98%. General anesthesia was induced with ketamine (8 mg), rocuronium (8 mg), and fentanyl (15 μg) with 100% oxygen under standard monitoring. After uneventful endotracheal intubation with a 3.0 mm Microcuff tube®, general anesthesia was maintained with sevoflurane (0.7–1.0%), midazolam (0.1 mg/kg/h), fentanyl (15 μg), remifentanil (0.1 μg/kg/min), and rocuronium (12 μg/kg/min). Following cefazolin (220 mg) administration, a radial arterial line and central venous catheter were placed via the right jugular vein. In total, 50 mL of 5% albumin was infused repetitively to treat mild hypotension (57/35–67/41 mmHg) and desaturation (SpO_2_ 85–87%).

We noticed the generalized erythema immediately after peeling away the drape for the central venous catheter cannulation. Shortly thereafter, the patient became profoundly hypotensive, with his systolic blood pressure dropping from 79/45 mmHg to 39/23 mmHg as measured through arterial line monitoring without any respiratory symptoms, such as wheezing, elevated airway pressure, or desaturation. Anaphylaxis was diagnosed, and the rocuronium, heparin, and 5% albumin administration were stopped. In total, 3.0 μg/kg of intravenous epinephrine was given. Continuous intravenous epinephrine and vasopressin infusion was started at 0.2 μg/kg/min and 0.15 mU/kg/min, respectively. In addition, methylprednisolone (28 mg/kg) and acetated Ringer’s solution containing 1% glucose (13 mL/kg) were given. At 40 min after onset, the generalized erythema and hypotension improved until a bolus injection of epinephrine was no longer required to maintain systolic blood pressure (> 75 mmHg). At 120 min after onset, the surgery was canceled, and the patient was transferred to the pediatric intensive care unit (PICU) with continuous epinephrine (0.15 μg/kg/min) and vasopressin (0.7 mU/kg/min) administration.

**Fig. 1:**
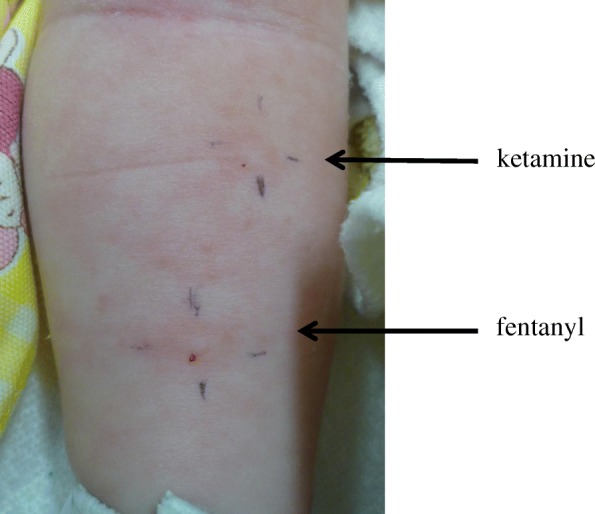
Positive flare response to intradermal test

Upon admission to the PICU, his blood pressure and HR were 97/44 mmHg and 145 bpm, respectively. He was intubated and sedated with midazolam (0.08 mg/kg/h) and morphine (0.8 mg/kg/day), and his hemodynamics stabilized with administration of dopamine (3 μg/kg/min) throughout his PICU stay. On postoperative day 3 (POD 3), intradermal testing was performed by pediatric allergologists for all the drugs given during the anaphylactic event. The drug concentrations for the skin test were based on previous studies [5] (Table 1). An allergologist performed an intradermal test on the forearm in the presence of the pediatric intensivists in the PICU. Fentanyl citrate induced an 11 × 6-mm flare response, and ketamine induced a 3 × 3-mm flare response (Fig. 1.). A positive reaction (wheal or flare) was not noted for the other drugs.

**Table 1:** Intradermal test for the drugs given during the anaphylactic event

Drug	Undiluted concentration (mg/mL)	Dilution	Wheal diameter (mm)	Erythema diameter (mm)
Control (saline)			0	0
25% albumin		1/10	0	0
Heparin		1/10	0	0
Rocuronium	10	1/100	0	0
Cefazolin		1 mg/mL	0	0
Fentanyl	0.05	1/10	0	11 × 6
Remifentanil		0.005 mg/mL	0	0
Ketamine	10	1/10	0	4 × 4

*SPT* skin prick test, *IDT* intradermal test

For the second operation scheduled on POD 5, anesthesia was maintained uneventfully with sevoflurane (0.7–1.0%), morphine (0.1 mg/kg bolus followed by 0.01–0.02 mg/kg/h infusion), and rocuronium (0.5 mg/kg/h). Fentanyl and remifentanil were not used. Administration of cefazolin and heparin did not cause anaphylaxis during the second surgery. Before administering the second anesthesia, we obtained the parent’s informed consent for the use of morphine as an alternative analgesic based on its chemical dissimilarity with fentanyl and its safety record in the PICU.

On POD 186, the patient’s postoperative cardiac catheter examination was scheduled. General anesthesia was induced with inhalational oxygen, nitrous oxide, and sevoflurane. Intubation was facilitated by morphine and rocuronium, and general anesthesia was maintained with sevoflurane and rocuronium. Sugammadex was used for muscle relaxant reversal without any adverse events. The examination was concluded without event.

## Discussion

Perioperative anaphylaxis is a life-threatening clinical condition. The agents responsible for anaphylaxis during anesthesia are muscle relaxants, antibiotics, latex, and plasma substitute in the order of frequency [2, 3]. In contrast, opioids very rarely cause anaphylaxis [6]. In our case, we were able to manage the patient, who showed hypersensitivity to fentanyl during the first anesthesia, by using morphine as an alternative opioid during the subsequent anesthesia.

For patients with clinical signs suggesting anaphylaxis, laboratory examinations, including tests for serum histamine and tryptase concentrations and skin tests, such as the prick test and intradermal test, are useful for a definitive diagnosis although some studies have reported that more than 95% of cases can be diagnosed on the basis of the clinical signs alone [7]. The serum tryptase concentration has a sensitivity of 64% and a specificity of 89.3% when the blood sample is drawn within 2 h after onset [4, 8]. In the present case, our pediatric allergologist clinically diagnosed anaphylaxis and decided not to assess the serum tryptase concentration due to the time and cost required. Finally, an intradermal test revealed that fentanyl citrate was the causative agent based on the criterion of the presentation of erythema with a minimum diameter of 8 mm [3].

Opioid anaphylaxis is usually classified as a non-allergic form of anaphylaxis [9]. However, the presence of a flare response to fentanyl citrate indicated allergic anaphylaxis despite the lack of any history of fentanyl use in our patient. Unfortunately, his initial exposure to the allergen (fentanyl or related substance) was not confirmed, and further research needs to be done to clarify the sensitization mechanism.

In the present case, intradermal testing was performed on POD 3 because the patient’s medical condition required surgery as soon as possible although intradermal testing is normally recommended 4–6 weeks after an anaphylactic event to prevent increasing the risk of false negative results [3, 6]. When performed earlier (within 1 week after an event), only the positive results should be taken into account [10]. For the second anesthesia on POD 5, morphine chloride was used as an alternative analgesic to fentanyl due to the difficulty of stabilizing the hemodynamic status of a patient with anoxic spells without using any opioids. Remifentanil was not used for subsequent anesthesia due to the possibility of a false negative result despite the negative intradermal test finding. Moreover, although both fentanyl and remifentanil are phenylpiperidine derivatives and cross-reactivity is uncommon among this group of substances [11], the possibility of such cross-reactivity should nonetheless be born in mind. Our allergologist decided not to perform a prick test prior to the intradermal test due to the smallness of the area on the patient’s forearm being used for a variety of skin tests. The allergologist performed an intradermal test in the presence of the pediatric intensivists and under intensive monitoring, including invasive blood pressure monitoring, in the PICU because the risk of a severe anaphylactic reaction caused by the skin tests was not able to be ruled out [12]. Morphine sulfate was used as an alternative analgesic for subsequent anesthesia because it is chemically dissimilar to phenylpiperidine derivatives and had been used without any adverse reaction in the PICU.

## Conclusions

Perioperative anaphylaxis is a rare event but can lead to life-threatening systemic allergic reactions. Opioids very rarely cause anaphylaxis. We experienced a case of anaphylaxis associated with a sudden onset of profound hypotension and generalized erythema after uneventful anesthesia induction. Intradermal testing revealed that fentanyl was the causative agent. We were able to manage the patient by using morphine as an alternative opioid during the subsequent anesthesia.

## Abbreviations

BTS: Blalock-Taussig shunt
FISH: Fluorescence in situ hybridization
PICU: Pediatric intensive care unit
POD: Postoperative day
SpO_2_: Oxygen saturation of peripheral artery
TOF: Tetralogy of Fallot
